# A Comparison of the Satiety Effects of a Fruit Smoothie, Its Fresh Fruit Equivalent and Other Drinks

**DOI:** 10.3390/nu10040431

**Published:** 2018-03-30

**Authors:** Peter J. Rogers, Roya Shahrokni

**Affiliations:** 1 Nutrition and Behaviour Unit, School of Experimental Psychology, University of Bristol, Bristol BS8 1TU, UK; roya.shahrokni@bristol.ac.uk; 2 National Institute for Health Research Bristol Biomedical Research Centre, University Hospitals Bristol NHS Foundation Trust and University of Bristol, Bristol BS8 1TU, UK

**Keywords:** energy-containing drinks, fruit smoothie, eating rate, fullness, energy intake compensation, liking, eating satisfaction

## Abstract

Energy-containing liquids are claimed to have relatively low satiating power, although energy in liquids is not without effect on appetite. Using the preload test-meal method, effects on fullness and energy intake compensation were compared across four drinks (water, blackcurrant squash, milk and fruit smoothie) and the fresh fruit equivalent of the smoothie. Preload volumes were similar, and the energy value of each preload was 569 kJ, except for water (0 kJ). Healthy, adult participants rated the preloads for liking, enjoyment, satisfaction, familiarity and how ‘food-like’ they seemed. The preload to test-meal interval was 2 min (*n* = 23) or 2 h (*n* = 24). The effects of the preloads on fullness varied with food-likeness and the rate at which they were consumed. In contrast, energy intake compensation versus water did not differ between the energy-containing preloads, although it decreased over time (from 82% at 2 min to 12% at 2 h). In conclusion, although fullness increased with food-likeness, subsequent energy intake compensation did not differ for energy/nutrients consumed in drinks compared with a food. The results also support the proposal that food intake is influenced predominantly by the immediate, but rapidly waning, post-ingestive effects of the previous ‘meal’ (rather than by changes in energy balance).

## 1. Introduction

Generally, perhaps with the exception of soup, energy-containing liquids are considered to be less satiating than foods, including their near equivalent semi-solid or solid foods (e.g., [1,2,3,4,5]). Furthermore, energy in a liquid has been found to have a greater satiating effect if the product is presented as a filling snack rather than as a thirst-quenching drink [6], and quite small differences in the oro-sensory properties of energy-containing liquids (e.g., ‘creaminess’) can apparently have a similar effect [7]. Other studies, though, have found no difference in the satiating effects of disguised nutrient (energy) loads provided in liquid, semi-solid and solid forms [8,9,10]. The explanation for these different results is unclear. Nonetheless, it is certain that energy in liquids (e.g., sugars) does affect appetite. This is demonstrated, for example, by the many studies that have compared ad libitum energy intake following the consumption of sugar-containing drinks versus oro-sensory matched, low/zero-energy sweet drinks (reviewed in [11]). It has been shown that, overall, there is a reduction in energy intake on the next eating occasion that compensates for half of the energy consumed in the sugar-containing drink [11]. The question, then, is to what extent (if any) is the satiating effect of energy greater in foods than in drinks? The importance of this question lies in informing the understanding of the control of appetite and energy intake, and in its implications for formulating interventions to improve weight management, ranging from public policy initiatives to individual dietetic practices.

Outside the laboratory, in everyday life, foods and drinks vary on many potentially important dimensions that might reasonably be expected to influence their satiating effects. An example is the rate of consumption—eating the same food more slowly reduces energy intake [12]. It is argued that liquid kJs are typically consumed faster than solid kJs, as the former can be gulped (i.e., consumed quickly) and the latter have to be chewed (e.g., [4]), although of course drinks can be sipped, and they are energy dilute compared to many foods (cf. [10]). An important determinant of the rate of consumption is the time the food or drink spends in the mouth (‘oral residence time’), which varies very substantially across different foods and drinks (e.g., [13]) in terms of g/min and less so in terms of kJ/min. At least part of the significance for this could be that food eaten more slowly is better remembered, which in turn increases fullness [14]. Based on differences in rate of consumption, it has even been suggested that liquid kJs ‘enter the body undetected’ [4]. This may be partly true for gulped liquids, but the energy consumed will be detected in the gut and post-absorptively and clearly does have a satiating effect as confirmed by the effects of sugar-containing drinks, outlined above [11]. Beyond eating rate, though, the same food in different forms, eaten at a fixed rate, has been shown to differ in its satiating effect per kJ (e.g., [3]).

The aim of present experiment was to compare the satiating effects of drinks varying in ‘food-like’ quality with the satiating effect of a food—fresh fruit—which was equivalent in energy content (569 kJ (136 kcal)) to the drinks. The same volume (250 mL) of water as the drinks provided a zero-energy control condition. We used the preload, test-meal method [15] to compare the satiating effect of consuming fixed portions of the different drinks and the fresh fruit (the preloads). The ad libitum test-meal comprised a savoury food and a sweet food of similar energy density, respectively cheese scones and chocolate-chip muffins. The primary outcome measures were the increment in fullness from before to immediately after consuming the preload, and energy intake compensation (i.e., difference in test-meal energy intake after each energy-containing preload versus water, expressed as a percentage of the energy content of the preload).

We were particularly interested in the satiating effect of the fruit smoothie and the extent to which this product was perceived to be drink-like versus food-like. Fruit smoothies, which comprise blended fresh fruit and fruit juice, are a relatively recent commercial innovation. The clearly food-like fruit salad preload contained the same fruits in the same portions as the fruit smoothie, and a blackcurrant-flavoured sugar-sweetened drink and cow’s milk comprised the other energy-containing preloads. We predicted that milk would be regarded as somewhat food-like, similar to the smoothie. We aimed for the experiment to be ecologically-relevant, so, for example, the rate of consumption was not controlled (i.e., it was left free to vary). Based on the research described above, we hypothesised that the more food-like and slower-consumed preloads would be more satiating. Energy intake compensation diminishes as time passes [5], presumably because physiological signals arising from the detection of the preload nutrients, and perhaps also the memory or salience of consuming the preload, wane over time [16]. Accordingly, we served the test-meal either 2 min or 2 h (separate groups of participants) after consumption of the preload, representing continuation of the ‘meal’ and a between-meals snack, respectively. We hypothesised that energy-intake compensation would be less after 2 h than after 2 min.

A further hypothesis was that the ratio of sweet to total test-meal food intake would be lower when the sweet preloads were consumed, predominantly the blackcurrant squash and the fruit smoothie, compared to water. This bears on the question as to whether consuming a sweet-tasting drink might help satisfy the desire for sweet food [10,11]. An alternative proposal is that sweet drinks increase the desire for sweetness (e.g., [17]).

## 2. Materials and Methods

### 2.1. Participants

Forty-eight healthy, non-dieting adults, aged 18–37 years, were recruited to start the experiment. Recruitment was via volunteer databases which consisted of members of the general public living in Bristol and students and staff at the University of Bristol. There were equal numbers of men and women, although one male participant randomised to the 2-min interval group failed to return for his final test session and was not rebooked or replaced because this occurred during the last scheduled week of testing for the experiment.

Exclusion criteria were (1) aged <18 or >40 years of age, (2) currently dieting, (3) veganism, (4) having a food ‘allergy’ or ‘sensitivity’, (5) being pregnant or breastfeeding, (6) having diabetes, (7) taking medicines that influence appetite, (8) smoking >5 cigarettes/week or equivalent, and (9) not willing to consume one or more of the test drinks and foods. Participants were rewarded with £50 on their completion of their final (i.e., fifth) test session.

All participants gave signed consent prior to starting the experiment. The experiment protocol was approved by the University of Bristol, Faculty of Science Human Research Ethics Committee (ref. # 1601174701).

### 2.2. Experiment Outline and Design

The experiment used the fixed preload, ad libitum test-meal (dependent variable) method [15]. The preloads (independent variable) were water, blackcurrant squash (a sugar-sweetened blackcurrant flavoured drink), cow’s milk, a fruit smoothie (i.e., a blended fruit and fruit juice drink) and the solid fresh fruit equivalent of the fruit smoothie. The non-water preloads were equi-caloric. The test-meal (dependent variable) consisted of a savoury food and a sweet food, namely cheese scones and chocolate-chip muffins. 

For one group of participants, the preload to test-meal interval was 2 min and for another group, it was 2 h. Within those groups, each participant received all five preloads. Participants were randomised to the 2-min and 2-h interval groups with the constraint that there would be equal numbers of men and women in both groups. Preload order was determined according to the same incomplete balanced Latin square design for each interval group. Because of the visible differences between the preloads, neither the experimenter (RS) nor the participants could be blinded to the preload manipulation. However, weighing of the test-meal foods before serving and after consumption was done without reference to the preload condition, and the experimenter was fully blind to the computerized collection of data on fullness, liking, etc., as well as time taken to consume the preload.

The primary outcome measures were the change in fullness from before starting to consume the preload to immediately before starting to consume the test-meal, and energy intake compensation in the test-meal. Secondary outcome measures were hunger, thirst and the proportion of sweet (chocolate-chip muffins) to total food consumed in the test-meal. We also compared the preloads on various characteristics evaluated by the participants, including taste pleasantness and the extent to which they were considered ‘food-like’, and the time taken to consume the preloads was measured.

Sample size (*n* = 48) was determined from mean differences and their standard deviations observed in previous studies [10,11,18]. We assumed 60% (335 kJ) compensation in the test-meal for the energy value of the nutrient-containing preloads versus water, which is higher than found for ‘disguised’ manipulations of the sugar content of a drink [11], but the manipulation of nutrient content in the present experiment was explicit (e.g., fruit smoothie versus water). On the other hand, as outlined in the Introduction, we predicted that compensation would be less in the 2-h than in the 2-min interval group. Based on an effect size of d = 0.55, the power for a 1-tailed test (we hypothesised that the energy-containing preloads would decrease energy intake compared to water) at α = 0.05 to reject the null hypothesis of no treatment effect was 85%.

### 2.3. Preloads and Test-Meal

Details of the preloads are shown in Table 1. The serving size of the drink preloads was 250 mL, which is the standard serving size for the fruit smoothie. The blackcurrant juice and milk preloads were formulated to have the same energy density as the fruit smoothie (i.e., 369 kJ/250 mL). The fruit salad preload was formulated to contain the same fruits in the same proportions (total weight = 233 g) as the fruit smoothie.

The preloads were prepared freshly during the morning of each test day, then covered and placed in a refrigerator for approximately one hour prior to the start of the test session. They were removed from the refrigerator 10 min before serving. The drink preloads were stirred within 2 min before serving. The temperature of the preloads when served was 12–15 °C. The drinks were served in a plain glass, and the fruit salad was served in a plain glass bowl with a fork. The preloads were accompanied by the following labels: ‘Water (spring water)’, ‘Blackcurrant Squash (with added sugar)’, ‘Milk (semi-skimmed and whole)’, ‘Fruit smoothie (apple, banana, orange, mango, passion fruit and peach)’, ‘Fruit Salad (apple, banana, orange, mango, passion fruit and peach)’.

The test-meal comprised cheese scones and chocolate-chip muffins, cut into bite-size pieces and served in separate plain glass bowls. As described elsewhere [10,15], serving the test meal foods in bite-sized pieces helps reduce ‘unit bias’, which is the tendency to consume all of a full piece (unit) of food, such as a whole biscuit or a whole muffin. Serving sizes were 208 g (2.93 MJ) for the cheese scones and 172 g (2.93 MJ) for the chocolate-chip muffins. Macronutrient content per 100 g of the cheese scones was 11.6 g protein, 16.0 g fat and 35.1 g carbohydrate, and for the chocolate-chip muffins, it was 5.0 g protein, 19.0 g fat and 52.0 g carbohydrate.

The drinks and foods used for the preloads and test-meal are all commonly available in the UK, and they were sourced from Sainsbury’s Supermarkets Ltd., London, UK. 

### 2.4. Measures

Energy intake in the ‘ad libitum’ test-meal was calculated by subtracting the weight of the bowl plus food at the end of the test-meal from the weight of the bowl plus food served and multiplying the result by the energy density of the food. This was done separately for the cheese scones and chocolate-chip muffins.

Using computerised, horizontal, 100 mm visual analogue scales, participants rated their current fullness (‘How full does your stomach feel right now?’), hunger (‘How hungry do you feel right now?’) and thirst (‘How thirsty do you feel right now?’). These scales were anchored on the left with the words ‘Not at all’ (=score of 0) and on the right with the word ‘Extremely’ (=score of 100) [14,19]. 

Participants also evaluated the preloads by responding to the following sets of questions: After taking a sip or bite of the preload they rated ‘How much do you like the taste of this drink/food right now?’ and ‘How strong is your desire to consume this drink/food right now?’ (anchored ‘Not at all’ = 0, ‘Extremely’ = 100), and they rated ‘What is the maximum you would be willing to pay for this drink/food if you were buying it for breakfast?’ (on a sliding scale incrementing in pence from 0 to £6.50) and ‘How like a drink or food is this product to you? (anchored ‘This is a drink’ = 0, ‘This is a food’ = 100). Immediately after consuming all of the preload, participants rated ‘How much did you enjoy consuming this drink/food?’ and ‘Overall, how satisfying did you find this drink/food?’ (anchored ‘Not at all’ = 0, ‘Extremely’ = 100). Previously, we have argued that, as assessed here, desire to eat is a measure of anticipated food reward, as is amount willing to pay, and enjoyment is a measure of experienced food reward, and satisfaction combines food reward and the fullness resulting from consumption [16,19]. 

Participants were given a maximum of 8 min to consume the preload, and a stopwatch visible to the participant was displayed on the computer screen. Participants pressed the F key on the computer keyboard immediately before starting and immediately after finishing their preload. These key presses started and stopped the stopwatch, and they were recorded by the computer programme, which enabled us to calculate the time taken to consume the preload. Participants were required to consume the entire portion of the preload.

On completion of their final test session, participants completed the restraint scale of the Dutch Eating Behaviour Questionnaire (DEBQ) [20] and the disinhibition scale of the Three Factor Eating Behaviour Questionnaire (TFEQ) [21], and they were weighed and their height was measured. Participants also rated their familiarity with the preload drinks and food. They responded to the question ‘How often do you consume this drink/food’? for each of the preloads, which were listed as ‘Water’, ‘Blackcurrant squash’, ‘Milk’, ‘Fruit smoothie’, ‘Fruit salad’. The response scale was ‘Daily’, ‘Weekly’, ‘Monthly’, ‘Every 2–3 months’, ‘Yearly’, ‘Less than once a year’ and ‘Never’, and responses were scored 7, 1, 0.25, 0.1, 0.02, 0.01 and 0, respectively, to provide an estimate of the number of times consumed per week. Lastly, participants completed a demand awareness question. They were asked ‘What is the experiment about? Please write down in no more than a few sentences what you think the purpose of this experiment is.’

### 2.5. Procedure

The experiment was presented as an investigation into ‘The effects of breakfast on appetite’. Testing took take place in the morning on weekdays. Participants were instructed to keep to their usual routine of physical activity, eating and drinking the evening before testing. As much as possible, participants attended their 5 test sessions over 5 weeks with one week between each session. Participants arrived for their test sessions after fasting overnight and received the preload as their ‘breakfast’. They were reminded at this point that at the end of the test session they would be asked not to eat again for the next 2 h. Participants were tested in groups of up to six, with each participant seated in a private booth within a larger room. The schedule of testing is summarised in Table 2. The interval between the end of the maximum time allowed for consumption of the preload (8 min) and the start of consumption of the test-meal was fixed (2 min for the 2-min interval group and 2 h and 2 min for the 2-h interval group). In reality, participants mostly finished consuming the preloads, especially the drinks, well within the 8 min allowed. When they were presented with the test-meal, participants were invited to eat ‘until you feel satisfied’.

### 2.6. Data Analysis

Data analysis was performed using IBM SPSS Statistics 23 (IBM Corporation, Armonk, NY, USA). First, we screened the data on test-meal energy intake for outliers. We calculated the total test-meal energy intake (cheese scones plus chocolate-chip muffins) for the water preload as the nominal control condition, minus the total energy intake for each of the other preloads and converted these data to *z*-scores. There were no *z*-scores lying outside of our criteria of *z* > 3.29 or *z* < −3.29 (i.e., no scores falling outside 99.9% of a normal distribution), and therefore, no participants were excluded from the data analyses.

As described elsewhere [22], we calculated individual energy intake compensation scores by subtracting test-meal energy intake after the energy-containing preload from the test-meal energy intake after the water preload, dividing by 569 kJ (the difference in the energy value of the energy-containing preload and the water preload) and multiplying by 100. We calculated change in fullness, hunger and thirst ratings by subtracting the ratings made before consumption of the preload (baseline) from the ratings made immediately before starting the test-meal. We calculated the ratio of sweet food to total food consumed in the test-meal (kJ chocolate-chip muffins consumed divided by kJ chocolate-chip muffins plus kJ cheese scones consumed).

We analysed the data on the characteristics of the preloads (liking, etc.) and time taken to consume the preloads using single factor (preload, 5 levels), repeated measures ANOVAs. Differences between all pairs of means were tested using the least significant difference (LSD) test. We analysed the energy-intake compensation scores using a two-way mixed factor analysis of variance (ANOVA) with preload (4 levels) being the within subject factor and preload to test-meal interval (2 levels) being the between subjects factor. The data on changes in fullness, hunger and thirst were analysed using two-way mixed factor ANOVAs, with preload (5 levels) being the within subject factor and preload to test-meal interval (2 levels) being the between subjects factor. We followed the two-factor analyses with single factor, repeated measures ANOVAs, conducted separately for the 2-min and 2-h interval groups, and then tested for differences between all pairs of means using the LSD test. Where appropriate, the Greehouse–Geisser correction was applied for effects involving preload, with corrected *p*-values reported. We recognise that the LSD test does not correct for multiple comparisons (family-wise error rate), but it has a high level of power to detect differences [23]. Accordingly, we interpreted the results of the LSD tests conservatively; that is, we describe and interpret patterns of results across the preloads (ordered from water to fruit salad) rather than contrasting all pairs of preloads.

## 3. Results

### 3.1. Participant Characteristics

Participants’ (*n* = 47) mean ± standard deviation (SD) age was 23.0 ± 4.5 years, their Body Mass Index (BMI) was 22.8 ± 4.7 kg/m^2^, their DEBQ restraint score (minimum and maximum possible scores are 1 and 5) was 2.33 ± 0.64, and their TFEQ disinhibition score (minimum and maximum possible scores are 0 and 16) was 7.45 ± 3.72. These results show that the participants were, on average, of a healthy weight and scored moderately low on dietary restraint and tendency toward disinhibited eating. The 2-min and 2-h interval groups were well matched on these characteristics (no more than a 5% difference between groups for any of these measures).

### 3.2. Participant Evaluations and Rate of Consumption of the Preloads

Table 3 shows that the preloads differed on a variety of characteristics evaluated by the participants, and particularly so in how food-like they were perceived to be and the rate at which they were consumed (time taken to consume). The fruit salad was rated as the most food-like and took over two-and-a-half times as long to consume as any of the other preloads. The fruit smoothie was rated as more food-like than the other ‘drinks’, although it took a similar time to consume, taking significantly longer only than water. Notably, the fruit smoothie and fruit salad were rated high on liking, desire to consume, enjoyment and satisfaction, and they were valued (amount-willing-to-pay measure) substantially higher than the other preloads. Participants were most familiar with water, followed by milk, and then blackcurrant squash, fruit smoothie and fruit salad which did not differ in regard to familiarity. As instructed, all of the participants consumed the entire portion of all of the preloads.

### 3.3. Primary Outcomes (Energy Intake Compensation And Fullness)

**Figure 1:**
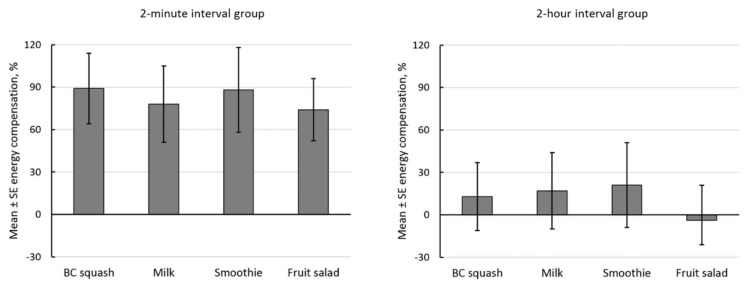
The data are mean and standard error (SE) energy-intake compensation scores (%) shown separately for the 2-min and 2-h preload to test-meal interval groups. They are the reduction in test-meal energy intake as a percentage of the energy content of the different energy-containing preloads (569 kJ) versus the water preload (0 kJ), Mean ± SD energy intakes after the water preload for the 2-min group and the 2-h group were respectively 2.62 ± 1.12 MJ and 2.98 ± 1.07 MJ. Compensation differed from zero (*p* = 0.002). There was no difference in compensation between the different preloads (*p* = 0.743). Compensation was greater in the 2-min group than in the 2-h group (*p* = 0.017). Mean compensation in the 2-min group was 82% and in the 2-h group it was 12%.

The results for energy intake compensation are summarised in Figure 1. Energy intake compensation differed from zero (intercept *F*(1,45) = 10.97, *p* = 0.002, partial *η*^2^ = 0.196), and it differed between the 2-min and 2-h interval groups (*F*(1,45) = 6.16, *p* = 0.017, partial *η*^2^ = 0.120). However, there was no effect of preload on energy intake compensation (*F*(3,135) = 0.41, *p* = 0.743, partial *η*^2^ = 0.009), nor was there a preload by interval interaction effect (*F*(3,135) = 0.09, *p* = 0.964, partial *η*^2^ = 0.002). There were no differences in energy intake compensation between preloads within the 2-min interval group (*F*(3,66) = 0.12, *p* = 0.947, partial *η*^2^ = 0.005) or within the 2-h interval group (*F*(3,69) = 0.49, *p* = 0.688, partial *η*^2^ = 0.021).

We analysed the change in fullness from before consumption of the preload (baseline) to immediately before consumption of the test-meal. Positive scores represent an increase in fullness. Baseline fullness (and hunger and thirst) was similar across the five preloads and similar for the 2-min and 2-h interval groups (Table 4, footnote). There was an effect of preload for change in fullness (*F*(4,180) = 18.86, *p* < 0.0001, partial *η*^2^ = 0.295), but no effect of interval (*F*(1,45) = 0.84, *p* = 0.363, partial *η*^2^ = 0.018) nor a preload by interval interaction effect (*F*(4,180) = 1.72, *p* = 0.147, partial *η*^2^ = 0.037). Table 4 shows that consumption of the preload led to an increase in fullness that was greatest for the fruit salad. Additionally, at 2 min after consumption, the increase in fullness did not differ between water, blackcurrant squash, milk and the fruit smoothie, whereas at 2 h after consumption, it was greater after milk and the fruit smoothie than after either the water or blackcurrant squash preload.

### 3.4. Secondary Outcomes (Hunger, Thirst and Proportion of Sweet Food Consumed in the Test-Meal)

For hunger, there was an effect of preload (*F*(4,180) = 15.67, *p* < 0.0001, partial *η*^2^ = 0.258), but no effect of interval (*F*(1,45) = 0.27, *p* = 0.608, partial *η*^2^ = 0.006), nor a preload by interval interaction effect (*F*(4,180) = 1.25, *p* = 0.292, partial *η*^2^ = 0.027). Essentially, the results for hunger were the inverse of those for fullness, with a similar pattern of differences between the preloads, showing that the fruit salad suppressed hunger the most and that the fruit smoothie and milk preloads had greater effects than water at 2 h after consumption (Table 4).

There was an effect of preload (*F*(4,180) = 17.95, *p* < 0.0001, partial *η*^2^ = 0.285) for thirst, but no main effect of interval (*F*(1,45) = 3.60, *p* = 0.064, partial *η*^2^ = 0.074), nor a preload by interval effect (*F*(4,180) = 0.94, *p* = 0.444 partial *η*^2^ = 0.020). Water, blackcurrant squash and milk reduced thirst the most, and fruit salad reduced thirst the least. The fruit smoothie had an effect on thirst intermediate between water, blackcurrant squash and milk, and the fruit salad (Table 4).

There was an effect of interval for the ratio of sweet to total food consumed in the test-meal (*F*(1,45) = 7.29, *p* = 0.010, partial *η*^2^ = 0.139). This ratio was lower for the 2-h interval group than for the 2-min interval group (Table 4). There was no effect of preload (*F*(4,180) = 0.97, *p* = 0.409, partial *η*^2^ = 0.021), nor a preload by interval interaction effect (*F*(4,180) = 1.92, *p* = 0.129, partial *η*^2^ = 0.041) for this measure. 

### 3.5. Demand Awareness

Based on their written responses to the demand awareness question, 20 (43%) participants were categorised as being unaware of the purpose of the experiment and 24 (51%) as being somewhat aware. Three participants (6%) were categorised as being fully aware—that is, they wrote that the experiment was testing the filling effects of the drinks and food or testing their effects on the amounts of scones and muffins consumed.

## 4. Discussion

Consistent with our hypothesis, the fillingness of the preloads (increase in fullness from before consumption of the preload to immediately before the start of the test-meal) varied in line with the extent to which they were perceived as food-like and the rate at which they were consumed. This relationship is particular striking in respect to the comparison between the fruit smoothie and the fruit salad. The latter was more filling, and it was consumed considerably more slowly and was rated as highly food-like. On the other hand, the fruit smoothie and fruit salad did not differ in regard to liking, desire to consume, enjoyment, satisfaction, familiarity or amount willing to pay. In other words, they were equivalent in terms of reward value, including monetary value. The smoothie and milk took longer to consume and were rated more food-like than water. They also increased fullness more than water, though this predominantly occurred two hours after consumption. Unexpectedly though, milk was rated less food-like than the fruit smoothie. This perception of milk may, at least in part, arise from its use in drinks such as tea and coffee. Overall, the results for hunger largely mirrored those for fullness. The extent to which the preloads reduced thirst tended to vary inversely with their food-likeness.

The pattern of results for test-meal energy intake differed in two ways from the pattern of results for fullness. First, test-meal energy intake was reduced equally by the energy-containing preloads (versus water), and second, it was reduced considerably more at 2 min compared to at 2 h after the preload (82% versus 12%). These results are consistent with an acute satiating effect of nutrients sensed in the gut during consumption and soon afterwards, but rapidly declining during the inter-meal interval [16,24]. In other words, it is to be expected that there will be little physiological ‘legacy’ 2 h after consuming 569 kJ. (It should be noted that for the 2-min interval group participants, 10 min elapsed between them starting to consume the preload and starting to consume the test-meal.)

The similar effect on test-meal energy intake of energy (sugar) in drinks and a food is consistent with results reported by Gadah et al. [10]. In that experiment, the preload to test-meal interval was 20 min, and energy intake compensation was 34%. In the present experiment, compensation was 82% for the 2-min interval group. However, this is for the comparison between the energy-containing preloads and water, rather than the sugar versus no-sugar (low-energy sweetener) preloads that were very similar in oro-sensory characteristics in Gadah et al. [10]. With obvious (i.e., explicit) differences between the various preloads and water, it may also be the case that participants, to some extent, consciously adjusted their test-meal intake to compensate for consuming ‘kJs’ in the energy-containing preloads. For example, it is possible that intake might have been adjusted according to the perceived unhealthiness/healthiness of the preload (which we did not measure), resulting in more conscious adjustment after the ‘added sugar’ blackcurrant squash drink than after the milk, fruit smoothie and fruit salad (cf. [25,26]). In turn, this might have been balanced by, for example, a greater satiating effect of the milk due to its relatively high protein content [27], and a greater satiating effect of the smoothie and fruit salad due to their fibre content [27] and/or the slower rate at which they were consumed. Whilst this is speculative, in another experiment that also compared the effects of explicitly different preloads, there was greater energy intake compensation for chocolate and sweet biscuit preloads than for equi-caloric fruit bars [28]. Indeed, there was ‘overcompensation’ (reduction in intake at lunch greater than the energy value of the preloads compared to no preload) for the chocolate and sweet biscuit preloads. The authors suggest that this may be explained by the chocolate and sweet biscuits being regarded by participants as relatively unhealthy. Alternatively, energy-intake compensation in the present experiment may have been driven primarily by the energy content of the preloads, with negligible effects of perceptions of healthiness and negligible enhancement of satiety by protein over carbohydrate or by fibre.

In contrast to energy-intake compensation, the pattern of effects observed for fullness may indicate that fullness was influenced primarily by cognition, and specifically, by the memory of what had been consumed as the preload [29,30]. The greater fillingness of the food (fresh fruit) compared to the drinks would then be explained by the expectation that foods are more satiating than drinks (cf. [31])—whilst energy intake compensation was affected predominantly by signals arising from the detection of nutrients in the gut and post-absorptively. The greater reported fullness 2 h after the more food-like drinks, milk and, more so, the fruit smoothie, than blackcurrant squash could be significant in everyday life, where timing of eating is free to vary (unlike the fixed times of the test-meal in this experiment). For example, greater fullness founded on the memory of having consumed a more food-like drink might cause a snack to be missed or a meal to be postponed. 

The present results differ from the lack of effect of viscosity on fullness observed by Gadah et al. [10], but in that experiment, the more food-like stimuli, namely blackcurrant jelly and blackcurrant candy, were smaller in volume than the drink (blackcurrant squash), as would be typical of such products in the world outside controlled laboratory studies. This probably resulted in these three preloads being consumed at similar rates in terms of kJs per minute. In the present experiment, both the volume and weight of the food (fresh fruit) and the volume and weight of the drinks were similar, because, like energy-containing drinks, fresh fruit has a low energy density. Consequently, consumption rate differed substantially between the fresh fruit, which required chewing before swallowing, and the drinks. Therefore, the longer oro-sensory exposure time associated with consumption of the fresh fruit may also help to account for its greater filling effect [4,13], perhaps at least in part because longer oro-sensory exposure leads to a stronger memory being formed of the consumption event [14]. To a lesser extent, this argument applies to the fruit smoothie, for which the rate of consumption was increased compared to water.

Some further points worthy of note include, first, the lack of relationship in this experiment between the fillingness of the preloads and the participants’ familiarity with the preloads. Previous research has found that the degree of familiarity with foods and also with eating foods to fullness increases their expected fillingness [32,33]. However, here, milk and blackcurrant squash, for example, differed substantially in how often they were consumed but had similar satiating effects. On the other hand, blackcurrant squash, fruit smoothie and fruit salad were equally relatively unfamiliar, but had substantially different filling effects. It seems likely, though, that whilst these drinks and the fruit salad were consumed relatively infrequently, they were by no means novel to participants, and furthermore, the accompanying labels identified them. Second, whilst the energy contents of the blackcurrant squash, fruit smoothie and fruit salad preloads were provided in the form of carbohydrate, predominantly as sugars, their fillingness differed, including between the fruit smoothie and the fruit salad which additionally had similar amounts of fibre. Third, in previous research, we argued that preload test-meal studies using within subjects designs probably underestimate energy compensation [18]. That, however, was in regard to disguised manipulations of, for example, the energy content of preloads. We would expect any underestimation effect to be smaller in studies where there are explicit differences between preloads, such as water versus fruit salad. In any case, compensation, at least when the test-meal followed 2 min after the preload, was 82% which, as noted above, is higher than observed in studies testing disguised differences in preload energy content [10,11]. 

A further hypothesis for the present experiment was that the relative amount of sweet food consumed in the test-meal would be reduced after consumption of the sweet (sugar-containing) preloads versus water [10,11,34]. Underlying this might be the generalisation of sensory-specific satiety [35] from the sweet preload to the sweet test-meal food (chocolate-chip muffin), or perhaps a purposeful reduction in chocolate-chip muffin intake based on balancing the sugar consumed in the preload. The latter might have been expected to apply particularly to the blackcurrant squash drink which was described (truthfully) as having ‘added sugar’. There was no evidence for such an effect, although it is also the case that neither was there evidence for an increase in the proportion of sweet food consumed after the sweet preloads versus water, which contradicts the proposal that consuming sweet drinks and foods engenders increased desire for sweetness (e.g., [17,36]). Notably, the relative amount of sweet food consumed was lower after the 2-h interval than after the 2-min interval. This was mainly due to an increase in intake of the savoury food—the cheese scones. This is not a result that we predicted but, as the 2-h interval group consumed the test-meal closer to lunchtime, it may reflect the greater ‘appropriateness’ [37] of cheese scones as a food for lunch than for breakfast, at least for UK consumers.

A limitation of this experiment is that no individual nutrient or cognitive variable, for example, viscosity, the rate of consumption or food-likeness, can be isolated as being solely responsible for the observed effects on fullness, hunger and energy compensation—that is, participants were not blind to differences between the preloads and the rate of consumption, liking, etc. differed between the preloads. Arguably, however, therein also lies one of its strengths, which is that it measured the effects of a combination of (measured) variables as present in ‘real’ rather than artificial, unfamiliar and/or improbable drink and food products (cf. [28]). Accordingly, it demonstrates potential roles of eating rate and food-likeness which, as discussed above, are to some extent at odds with claims based on more highly controlled (single-variable) studies. The present experiment also supports the advantage of consuming nutrient-rich products. For example, for the same cumulative energy intake (preload + test-meal), consumption of the fruit salad and the fruit smoothie compared to blackcurrant squash was associated with higher fibre and micronutrient intake. Additionally, the fruit smoothie was associated with greater enjoyment during consumption and greater fullness (after 2 h). A further strength of the experiment was that, despite the explicit differences between the preloads, very few participants guessed fully one or more of the hypotheses for the experiment. 

Lastly, it could be argued that measuring ad libitum energy intakes beyond the first post-preload meal would have revealed further compensation for preload energy intake. Previous research, however, suggests that this is unlikely. Levitsky and Pacanowski [38] found that participants ate 565 kJ more at lunch when they missed breakfast (2.62 MJ) compared to when they ate breakfast, but there were no further differences in energy intakes (total −21 kJ) in subsequent snacks and meals during the rest of the day. Similarly, in the present experiment, whilst test-meal energy intake was reduced 2-min after consumption of the energy-containing preloads compared to water, there was little compensation when the test-meal was delayed for 2 h. Very few studies have systematically varied the preload to test-meal interval in this way [16], but we suggest that this is an important observation in that it supports our proposal that food intake is influenced predominantly by rapidly waning post-ingestive effects of the previous meal rather than by signals related to short- or longer-term energy balance [24]. These effects will wane especially rapidly if the ‘meal’ is small, as was the case with the preloads we tested here. 

## 5. Conclusions

In summary and in conclusion, for a realistic set of drink and food stimuli, this experiment found that fullness increased with ‘food-likeness’. Thus, the fresh fruit salad was substantially more filling even than its equivalent drink—the fruit smoothie. By contrast, energy intake compensation did not differ between the fruit smoothie, the other drinks, or the fruit salad. However, energy intake compensation decreased markedly over 2 h. Together, these results suggest that, within typical meal patterns, energy consumed in a drink and energy consumed in a food can be expected to have similar effects on energy balance. The implication of these results for weight management is that irrespective of whether energy is consumed as food or drink, it is not fully compensated for by a reduction in energy intake at the next meal.

## Figures and Tables

**Table 1:** Nutrient content of the preloads.

	Protein, g	Fat, g	Carbohydrate, g ^a^	Fibre, g	kJ per Serving ^b^
Water ^c^	0	0	0	0	0
Blackcurrant squash ^d^	0.1	0	34.0	0	569
Milk ^e^	8.5	6.2	11.7	0	569
Fruit smoothie ^f^	1.1	0.3	30.0	3.5	569
Fruit salad ^g^	1.3	0.7	29.5	3.6	569

^a^ Of which sugars comprised 32.8 g for blackcurrant squash, 11.7 g for milk, 27.0 g for the fruit smoothie, and 28.8 g for the fruit salad. ^b^ For all preloads, the serving size was 250 mL, except for the fruit salad for which the serving size was 233 g. ^c^ Sainsbury’s still Scottish Mountain Water™ (Sainsbury’s Supermarkets Ltd., London, UK). ^d^ 63 mL Ribena™ blackcurrant concentrated squash (Lucozade Ribena Suntory Ltd., Stockley Park, Uxbridge, UK) diluted with 187 mL water. This squash is from the ‘dilutables’ category of soft drinks. It is a concentrated product for dilution by consumers according to their taste. ^e^ 165 mL semi-skimmed cow’s milk and 85 mL whole cow’s milk. ^f^ Magnificent mango fruit smoothie, Innocent Drinks™, London, UK. ^g^ Fresh apple (120 g), mango (38 g), banana (36 g), orange (27 g), passion fruit (7 g) and peach (5 g).

**Table 2:** Test session schedule.

Time	Activity	Measures
8.45 a.m.	Participant arrives at lab (and on first occasion, signs participant consent form).	Fullness, hunger and thirst.
8.58 a.m.	Participant tastes the preload.	Liking and desire to consume, amount willing to pay, and food-like versus drink-like.
9.00 a.m.	Participant consumes the preload.	Immediately after consumption: enjoyment and satisfaction. Time taken to consume the preload.
9.08 a.m.	2-min interval participants: ratings then wait. 2-h interval participants: Rest, allowed to read and/or study until 11.08 a.m.	Fullness, hunger and thirst, before the test-meal is presented (2-min interval participants only).
9.10–9.25 a.m.	2-min interval participants: Test-meal foods served (9.10 a.m.). Participants invited to eat until they feel ‘satisfied’. Remaining food removed after the participant indicates that they have finished eating.	Amounts of test-meal foods consumed (kJ). Ratio of sweet to sweet + savoury food consumed.
9.30 a.m.	2-min interval participants: Leave the lab. ^a^	
11.08 a.m.	2-h interval participants: ratings then wait.	Fullness, hunger and thirst, before the test-meal is presented.
11.10–11.25 a.m.	2-h interval participants: Test-meal foods served (11.10 a.m.). Participants invited to eat until they feel ‘satisfied’. Remaining food removed after the participant indicates that they have finished eating.	Amounts of test-meal foods consumed (kJ). Ratio of sweet to sweet + savoury food consumed.
11.30 a.m.	2-h interval participants: Leave the lab. ^a^	

^a^ Before leaving the lab on their final test day, participants completed the Dutch Eating Behaviour Questionnaire (DEBQ) [20] and the disinhibition scale of the Three Factor Eating Behaviour Questionnaire (TFEQ) [21], and their height and weight were measured. They were then debriefed and paid, and their consent was obtained for their data to be used.

**Table 3:** Characteristics of the preloads, rated by participants before and after consumption, and time taken to consume the preloads.

	Water	BC Squash	Milk	Smoothie	Fruit Salad	*F*(4,184), *p*
Liking, mm	60 ± 21 ^a^	60 ± 28 ^a^	59 ± 27 ^a^	79 ± 19 ^b^	75 ± 20 ^b^	8.24, <0.0001
Desire to consume, mm	64 ± 22 ^ab^	57 ± 27 ^a^	56 ± 26 ^a^	72 ± 19 ^c^	71 ± 20 ^bc^	5.78, =0.0002
Amount willing to pay, pence	66 ± 66 ^a^	94 ± 52 ^b^	84 ± 51 ^ab^	190 ± 88 ^c^	208 ± 80 ^c^	67.85, <0.0001
Food-like, mm	1 ± 2 ^a^	5 ± 14 ^b^	13 ± 18 ^c^	24 ± 21 ^d^	91 ± 13 ^e^	316.93, <0.0001
Enjoyment, mm	57 ± 26 ^a^	56 ± 30 ^a^	60 ± 28 ^ab^	81 ± 15 ^c^	69 ± 25 ^c^	9.43, <0.0001
Satisfaction, mm	48 ± 27 ^a^	56 ± 27 ^ab^	59 ± 24 ^b^	72 ± 19 ^c^	73 ± 21 ^c^	11.10, <0.0001
Time taken to consume, s	114 ^a^ ± 92	132 ^ab^ ± 117	133 ^ab^ ± 108	156 ^b^ ± 100	423 ^c^ ± 65	145.77, <0.0001
Familiarity, times consumed per week	6.8 ^a^ ± 1.0	0.8 ^b^ ± 1.9	3.6 ^c^ ± 3.3	0.7 ^b^ ± 1.7	1.0 ^b^ ± 2.1	77.36, <0.0001

BC Squash is Blackcurrant Squash. Data are means ± standard deviations (SD). Liking, desire to consume, enjoyment and satisfaction were rated on 0–100 mm scales, anchored ‘Not at all’ and ‘Extremely’. Food-like ratings were made on a 0–100 mm scale, anchored ‘This is a drink’ and ‘This is a food’. Means not sharing a superscript letter in common (^a, b, c, d^) differ significantly (*p* < 0.05, least significant difference (LSD) test).

**Table 4:** Summary statistics for fullness and the secondary outcome measures, shown separately for the 2-min and 2-h interval groups.

	Water	BC Squash	Milk	Smoothie	Fruit Salad	
2-min preload test-meal interval						*F*(4,88), *p*
Change in fullness, mm	17 ^a^ ± 19	18 ^a^ ± 19	23 ^a^ ± 15	25 ^a^ ± 21	37 ^b^ ± 22	4.95, =0.001
Change in hunger, mm	−6 ^a^ ± 20	−14 ^ab^ ± 21	−20 ^b^ ± 18	−15 ^ab^ ± 26	−31 ^c^ ± 22	5.34, <0.001
Change in thirst, mm	−44 ^a^ ± 28	−35 ^a^ ± 31	−37 ^a^ ± 35	−31 ^a^ ± 27	−11 ^b^ ± 20	5.90, <0.001
Ratio of sweet to total test-meal foods *	0.765 ^ac^ ± 0.209	0.746 ^ac^ ± 0.244	0.772 ^ac^ ± 0.197	0.711 ^ab^ ± 0.231	0.813 ^c^ ± 0.195	2.25, =0.094
2-h preload test-meal interval						*F*(4,92), *p*
Change in fullness, mm	10 ^a^ ± 14	17 ^a^ ± 24	29 ^b^ ± 21	31 ^b^ ± 23	48 ^c^ ± 20	15.74, <0.0001
Change in hunger, mm	4 ^a^ ± 19	−7 ^ab^ ± 17	−16 ^b^ ± 23	−21 ^bc^ ± 28	−34 ^c^ ± 29	11.16, <0.0001
Change in thirst, mm	−51 ^a^ ± 20	−53 ^a^ ± 18	−49 ^a^ ± 27	−35 ^b^ ± 24	−16 ^c^ ± 24	15.01, <0.0001
Ratio of sweet to total test-meal foods *	0.612 ± 0.227	0.583 ± 0.245	0.605 ± 0.237	0.629 ± 0.229	0.600 ± 0.215	0.52, =0.629

* kJ chocolate-chip muffins consumed divided by kJ chocolate-chip muffins plus kJ cheese scones consumed. Data are means ± SDs. Fullness, hunger and thirst were rated on 0–100 mm scales, anchored ‘Not at all’ and ‘Extremely’. Means not sharing a superscript letter (^a, b, c^) in common differ significantly (*p* < 0.05, LSD test). Mean ± SD values for baseline (pre-preload) fullness, hunger and thirst were 23 ± 10 mm, 72 ± 12 mm and 67 ± 18 mm for the 2-min group, and for the 2-h group, they were 20 ± 10 mm, 68 ± 19 mm and 73 ± 11 mm. Across the five preloads, mean baseline fullness varied between 20 mm and 23 mm, hunger varied between 69 mm and 72 mm, and thirst varied between 69 mm and 71 mm.
